# Does information on age-related fertility decline and fertility policies affect university students’ family and career expectations? Evidence from a randomized controlled trial

**DOI:** 10.1371/journal.pone.0287526

**Published:** 2023-11-01

**Authors:** Poh Lin Tan, Jessica Pan, Xing Xia

**Affiliations:** 1 Lee Kuan Yew School of Public Policy, National University of Singapore, Singapore, Singapore; 2 Department of Economics, National University of Singapore, Singapore, Singapore; 3 Division of Social Sciences, Yale-NUS College, Singapore, Singapore; University of Melbourne, AUSTRALIA

## Abstract

**Background:**

Past research shows that young adults have poor knowledge of age-related fertility decline and that the provision of information can improve fertility knowledge. We provide university students with information on age-related fertility and fertility-related policies and investigate whether the provision of such information affects their family formation and career expectations.

**Methods:**

A three-armed randomized controlled trial was conducted online in Singapore between September and October 2021. A total of 1000 undergraduate students were recruited through campus advertisements to complete a 30- to 45-minute online survey, which randomly exposed participants to one of three informational brochures on age-related fertility decline, fertility policies, or diabetes (control group). Participants answered questions on family formation and career expectations both before and after the information intervention. Analysis of covariance was used to assess the effects of the information intervention.

**Results:**

Exposure to age-related fertility information resulted in significant reductions in the ideal age at first childbirth, significant increases in the expected probability of marriage before age 30, and (among female participants) significant increases in the expected likelihood of undergoing social egg-freezing. No difference existed in child-number ideals, educational aspirations, and income expectations between groups after exposure. No difference existed between the fertility policy information group and the control group after exposure in any of the outcomes of interest.

**Conclusions:**

Information on age-related fertility decline brought forward university students’ expected timing of childbearing and marriage without reducing their educational and career expectations. The provision of fertility information at early ages, such as during university, can help correct widespread inaccurate beliefs about fertility and promote realistic family formation planning without adversely affecting educational and career goals.

**Trial registration:**

ClinicalTrials.gov.

## Introduction

Delayed childbearing has increased in prevalence in developed countries despite the risks of infertility and adverse reproductive outcomes associated with advanced maternal and paternal age [1–4]. However, many studies have shown that reproductive-aged populations generally have poor knowledge of age-related fertility decline [5–9], which may contribute to sub-optimal fertility choices and even involuntary childlessness [10–12]. Moreover, individuals, including healthcare professionals, tend to hold unrealistically optimistic beliefs regarding the ability of assisted reproductive technologies to overcome age-related infertility [6, 13–15].

Past studies suggest that informational interventions can help to address these knowledge gaps [16]. Several studies using randomized controlled trials show that exposure to evidence-based fertility information could improve fertility knowledge [17–20] and change the desired timing of childbearing in the short term [21]. We hypothesize that, in the East Asian context, exposure to fertility information could also affect the ideal age of marriage due to very low level of nonmarital childbearing in East Asian countries [22]. Moreover, no study has explored whether exposure to fertility information negatively affects participants’ educational and career expectations. Yet the potential effects of fertility education on young adults’ educational and career aspirations are important as family formation plans are often made simultaneously with career plans while considering both biological fertility constraints and financial concerns [23–25]. Both men and women consider a stable job and a dependable income as prerequisites for parenthood [11–13, 26]. Well-educated individuals often cite career planning as a key reason for delaying childbearing [25, 27, 28]. Relatedly, research shows that policies that improve the affordability of assisted reproductive technologies can influence a woman’s likelihood of obtaining a professional degree and choosing a professional career [29].

Moreover, although existing research suggests that young adults have poor knowledge about the legal rules and policies regulating assisted reproduction [30], previous intervention studies have focused exclusively on age-related fertility decline. Hence, the impact of providing assisted reproductive technologies-related policy information on family formation intentions is currently unknown.

### The present study

The primary aim of the present study was to examine the effects of providing fertility information and objective fertility policy information on university students’ family formation and career expectations. A total of 1,000 university students were randomly assigned to one of three treatment arms receiving information on age-related fertility (ARF group), fertility policies (FP group), or diabetes (control group). It was hypothesized that the provision of age-related fertility information would lead to forward adjustments in marriage and fertility plans (e.g., younger ideal ages at marriage and first childbirth and increased likelihood of undergoing social egg freezing), but lower educational and career expectations (e.g., lower chances of completing an advanced degree and lower future income). It was also hypothesized that the fertility policy information would increase the ideal number of children by lowering the perceived cost of having children and, at the same time, increase the expected likelihood of egg-freezing by highlighting the IVF subsidies. On the other hand, it was hypothesized that fertility policy information would have ambiguous effects on the expected timing of marriage and fertility (for example, information on IVF subsidies could induce individuals to postpone marriage and childbearing, whereas information on pro-natalist policies could incentivize early marriage and childbearing).

## Materials and methods

### Ethical approval

The study protocol was approved by the Institutional Review Board of the National University of Singapore (NUS-IRB-2020-362) prior to commencement. Study participants were presented with the Participant Information Sheet and Informed Consent Form at the beginning of the online survey. Participants provided consent by checking a box at the end of the Consent Form. Only those who provided consent were presented with the survey questionnaires and the intervention material. The consent process was approved by the ethics committee. Several pieces of personal information were collected from consenting participants, including name, student ID, email address, and phone number. These personal data were collected, processed, and stored by NUS Institute for Applied Learning Sciences and Educational Technology (ALSET) in accordance with University’s Research Data Management Policy. The authors received an anonymized copy of the survey responses with all identifiable information removed. None of the authors had access to participants’ names, student IDs, or email addresses during or after data collection. The first author (PLT) had access to students’ phone numbers for the purpose of administering the $15 health voucher; however, the phone number data were never linked to the anonymized survey responses and were used solely for administering the voucher. These protocols regarding privacy and data confidentiality were included in the Participant Information Sheet and approved by the ethics committee.

### Participants

Participants were undergraduate students at the National University of Singapore. Inclusion criteria were: full-time undergraduate student, aged between 20 and 24, unmarried, childless and not currently expecting, self-identifies as heterosexual, and Singaporean citizen. Participants were recruited in September and October 2021 via online advertisements posted in student clubs and a student research recruitment portal. The study was advertised as the “Youth Outlook on Life Opportunities Study” to reduce self-selection into the study on the basis of a particular interest in fertility-related issues. However, participants were informed before the start of the online survey that the questionnaire included questions on dating history and fertility issues. Participants were aware that they could withdraw from the study at any time. At the end of the survey, participants who completed the survey received a S$15 health voucher as a token of appreciation. The online survey was only accessible using the student’s unique student ID and allowed only one attempt from each ID, which ensured that the data collected pertained to 1,000 unique participants.

**Fig 1:**
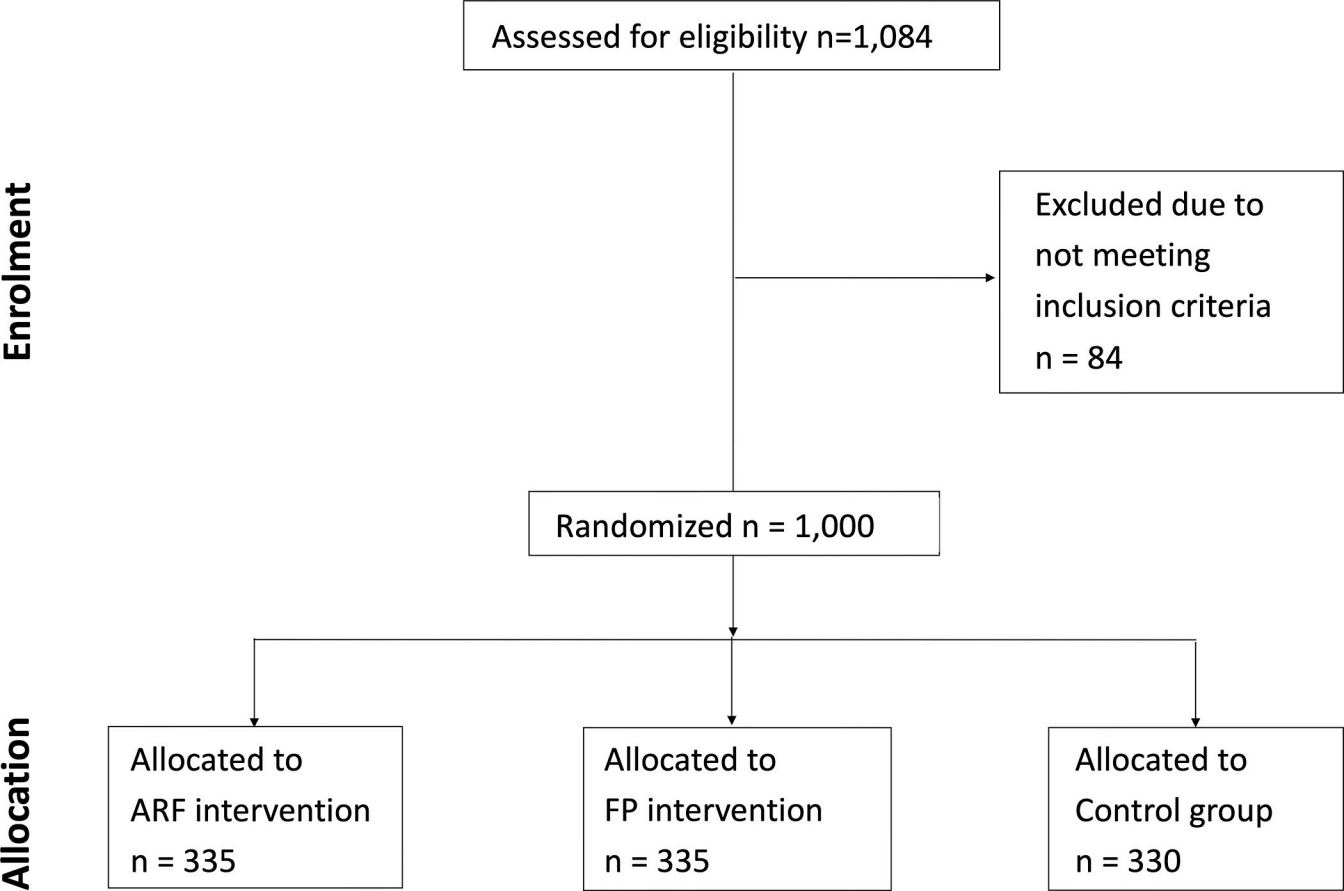
CONSORT flow diagram.

A total of 1,084 participants initiated the survey. Of these, 84 did not meet the inclusion criteria and did not participate in the survey or receive the information intervention. The remaining 1,000 participants completed the survey and were randomized into the three treatment arms. Fig 1 shows the participant flowchart. Due to the recruitment method described above, it was not possible to determine the number of eligible students who were exposed to the online advertisement. As a result, the precise response rate to this study cannot be determined. However, at the time of the experiment, there were 19,839 students that met our three primary inclusion criteria (full-time undergraduate student, between the ages of 20 and 24, Singaporean citizen). The lower bound of the response rate is 5.04% (calculated based on the assumption that all eligible students were exposed to the advertisement).

### Procedure

Participants who met the inclusion criteria completed an online survey that took 30–45 minutes. The online survey consisted of a pretest questionnaire, a one-page information sheet with the intervention material, and a posttest questionnaire. The survey and intervention material were presented online using Qualtrics. Participants completed the pretest questionnaire, were then randomized to receive one of three information interventions (ARF, FP, or control) and then completed the posttest questionnaire. Participants were asked to read the information presented thoroughly. Participants were not informed about how many information interventions existed during the online survey.

### Intervention

The ARF treatment arm was exposed to a six-item (164 words) informational sheet on age-related fertility decline and IVF effectiveness developed by the authors. In particular, the intervention material included information on IVF success rates for different age groups and statements such as “age is a more important predictor of women’s fertility than lifestyle choices” and IVF “success rates are higher for women who froze their eggs prior to age 35.”

The FP treatment arm was exposed to a six-item (219 words) educational information sheet on pro-fertility policies in Singapore, including subsidies and regulations related to assisted reproductive technologies, baby bonuses, and housing and childcare subsidies for young couples. The control group was exposed to a six-item (160 words) information sheet on diabetes, which included information on diabetes risk factors, treatment, and related policies.

Intervention materials are listed in S1 File. To develop the content of the brochures, existing literature [31, 32] and statistics from websites of local hospitals, fertility clinics, and the Ministry of Health were used.

### Measures

The pre- and posttest surveys consisted of 72 items that covered five domains developed to examine factors related to participants’ family and career expectations. Those questions relevant to the analyses presented in this paper are described here.

### Data collected pretest only

Background characteristics of participants were collected pretest, including age, year of study, area of study, gender, ethnicity, mother’s and father’s highest level of education, number of siblings, current relationship status (single, in a relationship), number of past relationships, plans to have children (yes, no, unsure), and plans to get married (yes, no, unsure).

### Data collected both pre- and posttest

Family formation and career expectations were collected both pre- and posttest. Questions on family formation expectations included ideal age at first birth, probability of having at least one child by age 30 (0 to 100), ideal number of children, ideal age at marriage, probability of getting married by age 30 (0 to 100), and probability that future spouse is 30 or younger at the time of marriage (0 to 100). Additionally, female participants also stated the expected probability that they would undergo social egg-freezing by age 25 and by age 30 (0 to 100). Questions on career expectations included probability of obtaining a master’s degree (0 to 100), probability of obtaining a doctoral degree, and expected annual income at age 30.

### Data collected posttest only

Information evaluation and psychological measurement was taken once immediately after exposure to the information brochure. We used a single item to evaluate the usefulness of the information brochure (rated on a 5-point Likert scale): ‘How do you feel about the information just presented?’ ‘I found the information useful: 1-Strongly disagree; 2-Disagree; 3-Neither agree nor disagree; 4-Agree; 5-Strongly agree.’ We used a single item to examine anxiety [19]: ‘How do you feel about the information just presented?’ ‘I feel anxiety: 1-Strongly disagree; 2-Disagree; 3-Neither agree nor disagree; 4-Agree; 5-Strongly agree.’

### Statistical analysis

Pretest demographic characteristics were summarized overall and by treatment arm. Differences across treatment arms were compared using ANOVA tests (for continuous variables) and *χ*^2^ tests (for categorical variables). Analysis of covariance (ANCOVA) was used to assess the effects of the interventions on participants’ child-timing and marriage-timing ideals and educational and income expectations. For each ANCOVA analysis, a linear regression model was fitted with the posttest measurement of the outcome as the dependent variable, and a treatment group indicator and the pretest measurement of the outcome as independent variables. We also adjusted for gender to reduce the potential for confounding, as gender was not balanced between the treatment arms (Table 1) and exhibited some association with the outcome variables. This ANCOVA approach has been shown to have the greatest statistical power for RCTs with both baseline and follow-up measurements [33, 34]. Post hoc interaction tests were conducted to explore whether the effects of the interventions were heterogeneous by participants’ gender [34]. Expected income at age 30 was positively skewed and log transformed for parametric analysis. All statistical tests were 2-sided, with α = 0.05. Analyses were conducted using STATA, version 17.

**Table 1:** Pretest characteristics of the total sample and the intervention and control groups.

Characteristic	Total Sample		ARF Intervention		FP Intervention		Control		P value*
	*N* = 1,000		*n* = 335		*n* = 335		*n* = 330		
Age in years, mean (SD)	21.9	(1.17)	21.8	(1.13)	21.9	(1.12)	21.9	(1.25)	0.075^a^
Year of study, n (%)									
Year 1	95	(9.5)	29	(8.7)	33	(9.9)	33	(10.0)	0.658^b^
Year 2	259	(25.9)	89	(26.6)	84	(25.1)	86	(26.1)	
Year 3	322	(32.2)	114	(34.0)	110	(32.8)	98	(29.7)	
Year 4	274	(27.4)	92	(27.5)	86	(25.7)	96	(29.1)	
Year 5	50	(5.0)	11	(3.3)	22	(6.6)	17	(5.2)	
Area of study, n (%)									
Medicine or Dentistry	201	(20.1)	64	(19.1)	64	(19.1)	73	(22.1)	0.534^b^
Other	799	(79.9)	271	(80.9)	271	(80.9)	257	(77.9)	
Gender, n (%)									
Male	390	(39.0)	112	(33.4)	134	(40.0)	144	(43.6)	0.024^b^
Female	610	(61.0)	223	(66.6)	201	(60.0)	186	(56.4)	
Ethnicity, n (%)									
Chinese	932	(93.2)	312	(93.1)	311	(92.8)	309	(93.6)	0.640^b^
Indian	25	(2.5)	8	(2.4)	7	(2.1)	10	(3.0)	
Malay	19	(1.9)	9	(2.7)	6	(1.8)	4	(1.2)	
Other	24	(2.4)	6	(1.8)	11	(3.3)	7	(2.1)	
Mother completed university, n (%)									
Yes	395	(39.5)	139	(41.5)	122	(36.4)	134	(40.6)	0.358^b^
No	605	(60.5)	196	(58.5)	213	(63.6)	196	(59.4)	
Father completed university, n (%)									
Yes	460	(46.0)	146	(43.6)	156	(46.6)	158	(47.9)	0.522^b^
No	540	(54.0)	189	(56.4)	179	(53.4)	172	(52.1)	
Number of siblings, n (%)									
None	133	(13.3)	41	(12.2)	43	(12.8)	49	(14.8)	0.724^b^
One	517	(51.7)	179	(53.4)	179	(53.4)	159	(48.2)	
Two	265	(26.5)	86	(25.7)	83	(24.8)	96	(29.1)	
Three or more	85	(8.5)	29	(8.7)	30	(9.0)	26	(7.9)	
Current relationship status, n (%)									
Single	565	(56.5)	195	(58.2)	185	(55.2)	185	(56.1)	0.724^b^
In a relationship	435	(43.5)	140	(41.8)	150	(44.8)	145	(43.9)	
Past relationship experience, n (%)									
Never been in a relationship	361	(36.1)	123	(36.7)	115	(34.3)	123	(37.3)	0.702^b^
Have been in a relationship	639	(63.9)	212	(63.3)	220	(65.7)	207	(62.7)	
Plan to have children one day, n (%)									
Yes	600	(60.0)	185	(55.2)	207	(61.8)	208	(63.0)	0.234^b^
Unsure	294	(29.4)	108	(32.2)	93	(27.8)	93	(28.2)	
No	106	(10.6)	42	(12.5)	35	(10.4)	29	(8.8)	
Plan to get married one day, n (%)									
Yes	783	(78.3)	256	(76.4)	270	(80.6)	257	(77.9)	0.681^b^
Unsure	185	(18.5)	69	(20.6)	54	(16.1)	62	(18.8)	
No	32	(3.2)	10	(3.0)	11	(3.3)	11	(3.3)	

*Note*: Values are number (percentage) except for the first row, which shows the mean and standard deviation of age in years.

^a^ ANOVA test

^b^ χ2 test

* P values are based on ANOVA or χ2 tests of equality across the three groups.

## Results

### Participant characteristics and group equivalence

In total, 1,000 students were recruited, randomized into the treatment arms, and completed the study. Table 1 shows baseline demographic characteristics of the participants by treatment group. The majority of participants (86.7%) had at least one sibling. Less than half (43.5%) were in a relationship at the time of the survey; a greater share (63.9%) had been in a relationship. A small minority (10.6%) did not plan to have children; another 29.4% were unsure if they wanted children. The three treatment arms were generally well balanced, being equivalent on all demographic variables except that the ARF group comprises a higher share of female students than the other two groups (*χ*^2^(2) = 7.49, *P*<0.05).

### Effect of the intervention on outcomes

#### Child-timing and child-number expectations

The mean ideal age at first childbirth was 30.3 years at pretest (median = 30 years; interquartile range, 29 to 32; range, 21 to 40). Exposure to age-related fertility information significantly reduced the ideal age at first childbirth. At posttest, the average ideal age was lower in the ARF group than in the control group, with a crude between-group difference of 0.28 years (P = 0.155) and an adjusted difference of 0.25 years (P = 0.013) (after adjusting for pretest measures of the outcome variable and gender) (Table 2). Exposure to fertility policy information did not have any statistically significant impact on the ideal age at first childbirth. The posttest difference in ideal age between the FP group and the control group was small and statistically nonsignificant.

**Table 2:** Pre and posttest measures of family formation, educational, and income expectations.

Outcome Variable	Time	ARF		FP		Control		ARF vs Control		FP vs Control	
		Intervention		Intervention		Group		adjusted		adjusted	
		n	mean	n	mean	n	mean	difference^a^		difference^a^	
			(SD)		(SD)		(SD)	[95% CI]	P value	[95% CI]	P value
Ideal age at first child^b^	Pre	293	30.3	300	30.3	301	30.3	..	..	..	..
			(2.31)		(2.16)		(2.27)				
	Post	293	29.8	300	30.2	301	30.1	-0.25**	0.013	0.11	0.243
			(2.34)		(2.23)		(2.40)	[-0.45, -0.05]		[-0.08, 0.30]	
Probability of first child	Pre	293	47.7	300	47.2	301	48.4	..	..	..	..
by age 30^b^			(28.2)		(28.6)		(28.1)				
	Post	293	58.3	300	54.2	301	53.3	5.28***	0.003	1.65	0.339
			(30.1)		(30.1)		(30.6)	[1.78, 8.78]		[-1.73, 5.02]	
Ideal number of	Pre	335	1.8	335	1.9	330	2.0	..	..	..	..
children			(0.88)		(0.82)		(0.84)				
	Post	335	1.8	335	1.9	330	2.0	0.01	0.728	0.02	0.293
			(0.90)		(0.87)		(0.85)	[-0.02, 0.04]		[-0.02, 0.06]	
Ideal age at marriage^c^	Pre	325	28.2	323	28.1	319	28.2	..	..	..	..
			(2.19)		(2.06)		(2.00)				
	Post	325	28.2	323	28.3	319	28.4	-0.14	0.271	-0.10	0.291
			(2.89)		(1.98)		(2.15)	[-0.38, 0.11]		[-0.28, 0.08]	
Probability of marriage	Pre	325	57.6	324	63.4	319	59.5	..	..	..	..
by age 30^c^			(25.4)		(24.0)		(24.9)				
	Post	325	65.2	324	66.7	319	62.4	4.18***	0.002	1.33	0.281
			(25.8)		(23.7)		(24.9)	[1.52, 6.84]		[-1.09, 3.75]	
Probability spouse is 30 or	Pre	325	59.7	324	63.1	319	61.9	..	..	..	..
younger at marriage^c^			(25.8)		(24.8)		(25.1)				
	Post	325	66.0	324	65.4	319	63.6	3.93***	0.007	0.88	0.532
			(25.3)		(25.3)		(25.5)	[1.10, 6.76]		[-1.90, 3.66]	
Probability of egg-freezing	Pre	223	20.1	201	22.7	186	17.6	..	..	..	..
by age 25^d^			(31.5)		(33.5)		(29.6)				
	Post	223	24.1	201	21.1	186	16.5	5.53***	0.005	0.64	0.736
			(33.4)		(32.1)		(28.6)	[1.66, 9.41]		[-3.09, 4.37]	
Probability of egg-freezing	Pre	223	34.4	201	34.0	186	27.7	..	..	..	..
by age 30^d^			(32.9)		(34.0)		(30.0)				
	Post	223	39.6	201	34.4	186	28.1	6.57***	0.002	1.46	0.466
			(32.4)		(32.8)		(29.6)	[2.44, 10.71]		[-2.47, 5.39]	
Probability of obtaining	Pre	335	39.0	335	38.6	330	39.7	..	..	..	..
Master’s degree			(28.3)		(30.2)		(28.4)				
	Post	335	38.9	335	39.2	330	39.1	0.32	0.779	0.97	0.408
			(28.3)		(29.9)		(28.4)	[-1.92, 2.56]		[-1.33, 3.27]	
Probability of obtaining	Pre	335	16.8	335	16.7	330	19.7	..	..	..	..
Doctoral degree			(22.1)		(24.3)		(24.2)				
	Post	335	18.2	335	17.9	330	20.9	-0.19	0.855	-0.45	0.663
			(24.0)		(24.4)		(25.0)	[-2.20, 1.83]		[-2.47, 1.57]	
Expected income at age 30	Pre	335	11.0	335	11.0	330	11.1	..	..	..	..
in log scale			(0.37)		(0.39)		(0.36)				
	Post	335	11.0	335	11.0	330	11.0	-0.01	0.533	-0.02	0.442
			(0.36)		(0.42)		(0.40)	[-0.05, 0.03]		[-0.06, 0.03]	

^a^ Adjusted difference in means between the intervention and the control group from ANCOVA models adjusting for pretest measurement and gender. Point estimates (with 95% CI in brackets) are shown in each cell

*** p<0.01

** p<0.05

* p<0.1

^b^ Asked only of participants who answered "yes" or "unsure" to whether they planned to have children one day.

^c^ Asked only of participants who answered "yes" or "unsure" to whether they planned to get married one day.

^d^ Asked only of female participants.

**Fig 2:**
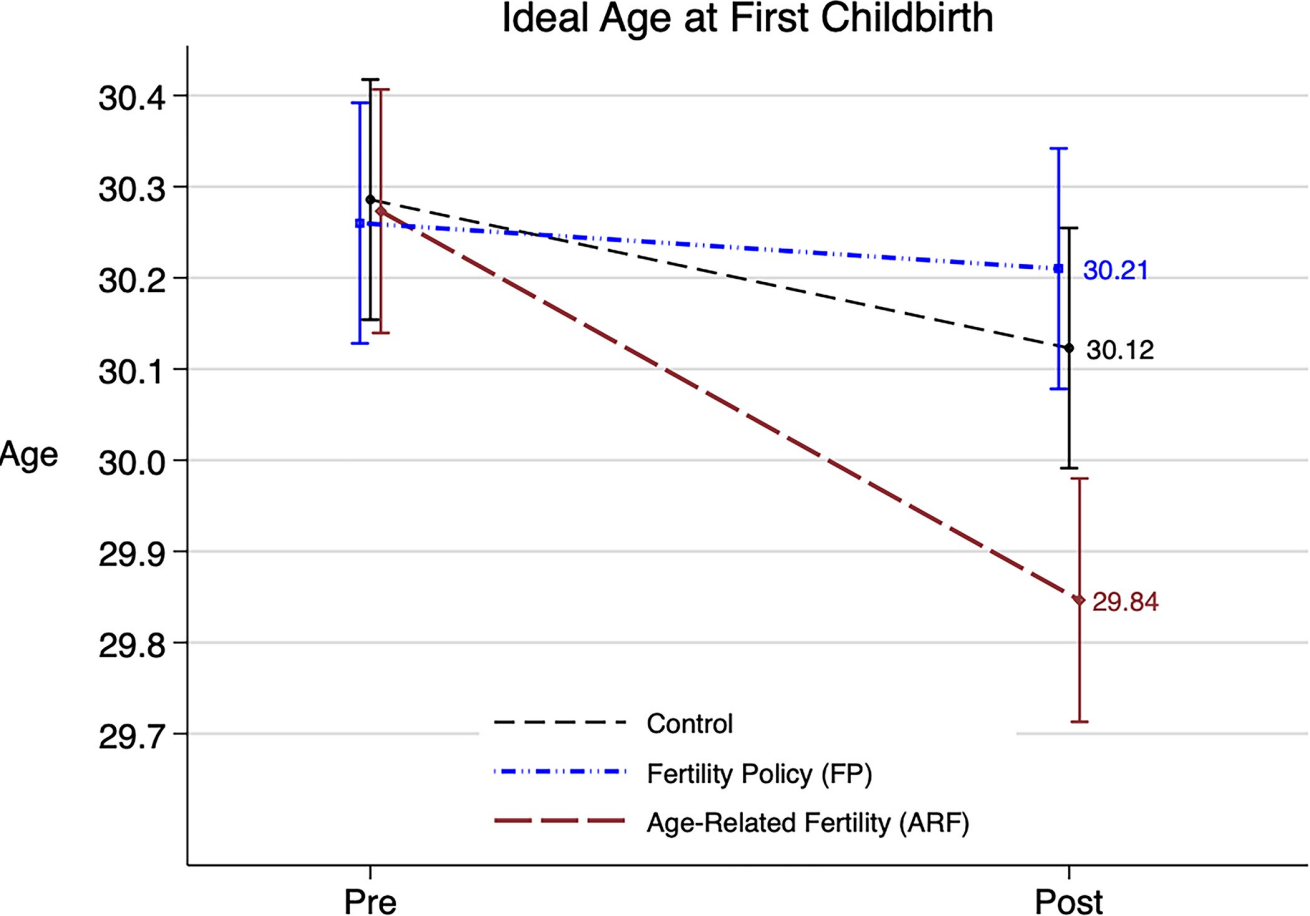
Pre- and posttest measures of ideal age at first childbirth by treatment group. ARF = Age-related fertility information intervention. FP = Fertility policy information intervention. Posttest measures are taken after exposure to the assigned intervention material. Pre- and posttest ideal ages at first childbirth are plotted at 1 standard error below and above the mean. Labels show the posttest mean ideal age at first childbirth for each treatment group. Errors bars represent standard errors.

Fig 2 illustrates the change in average ideal age at first childbirth before and after the intervention in each treatment arm. The average ideal age declined by 0.43 years in the ARF group (from 30.3 years at pretest to 29.8 years at posttest); the decline was statistically significant (P = 0.027). In contrast, the decline in average ideal age was small and statistically nonsignificant in both the FP group (change in mean age = -0.05 years; P = 0.780) and the control group (change in mean age = -0.16 years; P = 0.393).

**Fig 3:**
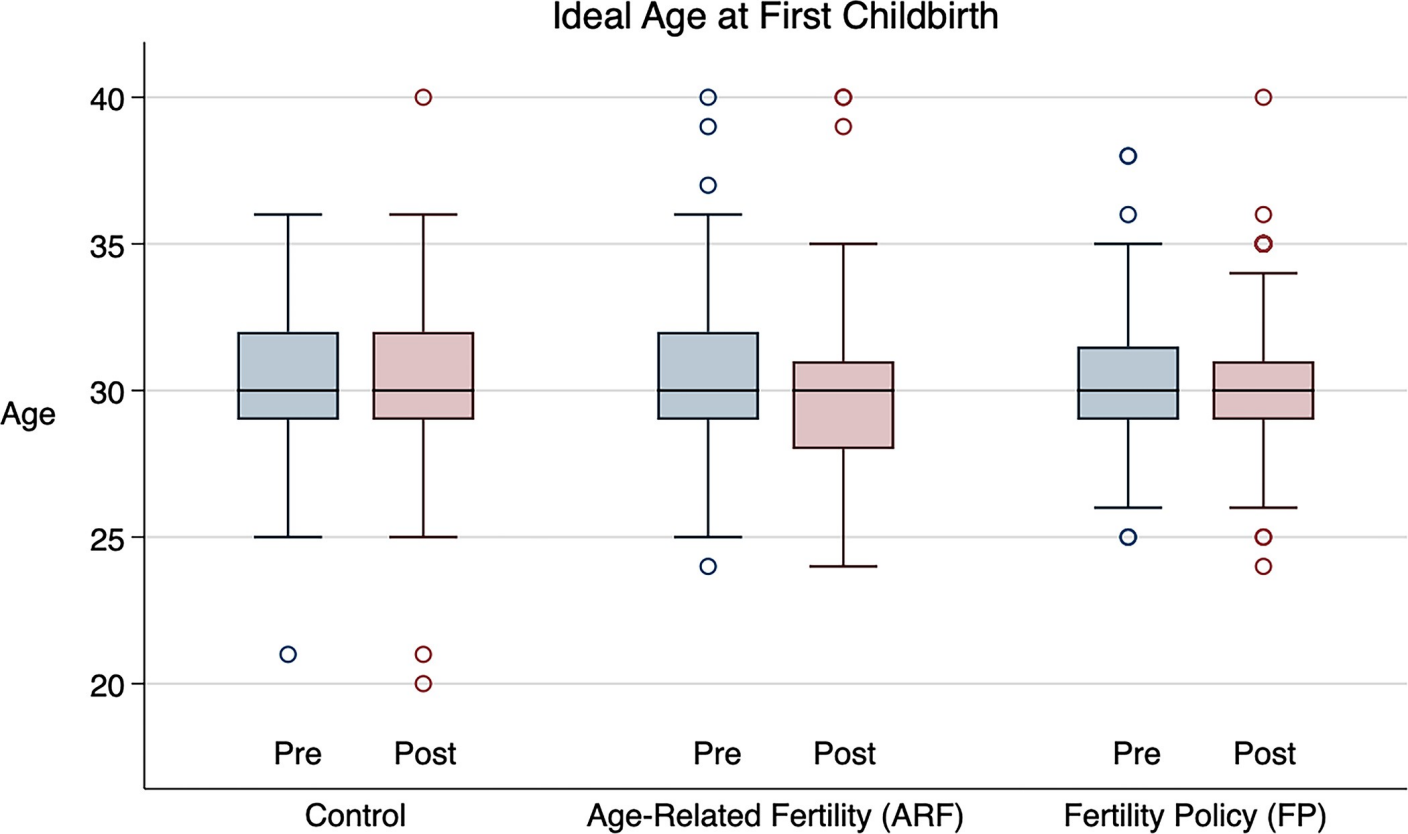
Box plot of pre- and posttest measures of ideal age at first childbirth by treatment group. Within each box, horizontal black lines denote median values. Boxes extend from the 25^th^ to the 75^th^ percentile of each group’s distribution of values. Whiskers extend to adjacent values (i.e., extreme values within a distance of 1.5 times the interquartile range from the nearest end of the box). Circles denote outside values (i.e., observations outside the range of adjacent values).

Fig 3 illustrates the change in the median and interquartile range of ideal age at first childbirth before and after the intervention in each treatment arm. In all three treatment arms, the median ideal age was 30 at pretest and did not change between pre- and posttest. In the control group, the interquartile range was 29 to 32 at pretest and did not change between pre- and posttest. However, in the ARF group, the interquartile range changed from 29 to 32 at pretest to 28 to 31 at posttest. In the FP group, the interquartile range changed slightly from 29 to 31.5 at pretest to 29 to 31 at posttest.

The mean expected probability of having a child by age 30 was 47.8 percent at pretest (median = 50; interquartile range, 25 to 70; range, 0 to 100). The age-related fertility information significantly increased participants’ expected probability of having a child by age 30. At posttest, the expected probability was significantly higher in the ARF group than in the control group, with an adjusted difference of 5.28 percentage points (P = 0.003) (Table 2). Exposure to fertility policy information did not have any statistically significant impact on the expected probability of having a child by age 30 (Table 2).

The mean Ideal number of children was 1.9 at pretest (median = 2; interquartile range, 2 to 2; range, 0 to 5). Neither the age-related fertility information nor the fertility policy information had a statistically significant impact on the ideal number of children (Table 2).

### Marriage-timing expectations

At pretest, the mean ideal age at marriage was 28.2 years (median = 28 years; interquartile range, 27 to 30; range, 20 to 40). Neither age-related fertility information nor fertility policy information had any statistically significant impact on the ideal age at marriage. The within-group change in ideal age at marriage pre- and post-intervention was small and statistically nonsignificant in all three groups (P = 0.714 for AFP group, P = 0.325 for FP group, and P = 0.127 for control). There were no differences between groups in ideal age at marriage at posttest (Table 2).

At pretest, the mean expected probability of marriage by age 30 was 60.2 percent (median = 60 percent; interquartile range, 50 to 80; range, 0 to 100). Exposure to age-related fertility information significantly increased participants’ expected probability of marriage by age 30. At posttest, the expected probability was significantly higher in the ARF group than in the control group, with an adjusted difference of 4.18 percentage points (P = 0.002) (Table 2). Exposure to fertility policy information did not have any statistically significant impact on the expected probability of marriage by age 30 (Table 2). Simple comparisons before and after the intervention also show that participants’ expected probability of marriage by age 30 increased significantly after the intervention in the ARF group (change in probability = 7.60 percentage points; P < 0.001). In contrast, the change in expected probability was small and statistically nonsignificant in both the FP group (change in probability = 3.36 percentage points; P = 0.073) and the control group (change in probability = 2.91 percentage points; P = 0.141).

At pretest, the mean expected probability that the participant’s spouse would be 30 or younger at the time of marriage was 61.6 percent (median = 60; interquartile range, 50 to 80; range, 0 to 100). Exposure to age-related fertility information significantly increased this expected probability. At posttest, the expected probability was significantly higher in the ARF group than in the control group, with an adjusted difference of 3.93 percentage points (P = 0.007) (Table 2). Exposure to fertility policy information did not have any statistically significant impact on this expected probability (Table 2). Simple comparisons before and after the intervention similarly show that the expected probability increased significantly in the ARF group (change in probability = 6.35 percentage points; P = 0.002). In contrast, the change in expected probability was small and statistically nonsignificant in both the FP group (change in probability = 2.29 percentage points; P = 0.245) and the control group (change in probability = 1.72 percentage points; P = 0.391).

### Expected probability of social egg-freezing

We analyzed the effects of the interventions on female participants’ expected chances of undergoing social egg-freezing by age 25 and age 30. At pretest, female participants’ mean expected probability of egg-freezing by age 25 is 20.2 percent (median = 0 percent; interquartile range, 0 to 30; range, 0 to 100); the mean expected probability of egg-freezing by age 30 is 32.2 percent (median = 22.5 percent; interquartile range, 0 to 50; range, 0 to 100). Exposure to age-related fertility information significantly increased female participants’ expected probability of undergoing social egg-freezing at both ages 25 and 30. At posttest, the expected probabilities were significantly higher in the ARF group than in the control group, with an adjusted difference of 5.53 percentage points for egg-freezing by age 25 (P = 0.005) and 6.57 percentage points for egg-freezing by age 30 (P = 0.002) (Table 2). Exposure to fertility policy information did not have any statistically significant impact on the expected probability of undergoing social egg-freezing by either age.

### Expected educational attainment and future income

For all three outcome variables measuring expected educational attainment and future income, the change over time was small and statistically nonsignificant in all three groups. At posttest, there were no differences between groups in any of the three outcome variables (Table 2).

### Information evaluation and post-intervention anxiety

On average, participants rated both the age-related fertility and the fertility policy information significantly more useful than the control information (both P < 0.001). Participants also rated the age-related fertility information marginally more useful than the fertility policy information (P = 0.060). The average ratings for usefulness of the information brochure were 4.13 in the ARF group (median = 4; interquartile range, 4 to 5; range, 1 to 5), 4.03 in the FP group (median = 4; interquartile range, 4 to 4; range, 1 to 5), and 3.80 in the control group (median = 4; interquartile range, 4 to 4; range, 1 to 5).

Participants’ posttest anxiety ratings were significantly higher in the ARF group than in the FP group (P = 0.002) and the control group (P < 0.001). The anxiety ratings were also significantly higher in the FP group than in the control group (P = 0.003). The average posttest anxiety ratings were 2.92 in the ARF group (median = 3; interquartile range, 2 to 4; range, 1 to 5), 2.67 in the FP group (median = 3; interquartile range, 2 to 3; range, 1 to 5), and 2.44 in the control group (median = 2; interquartile range, 2 to 3; range, 1 to 5).

### Subgroup analysis

We conducted post hoc analyses to explore whether the effects of the age-related fertility information on the primary outcome, the ideal age at first childbirth, were heterogeneous by participants’ gender. At pretest, the average ideal age at first childbirth was significantly lower among female participants (mean = 29.7; SD = 1.94) than among male participants (mean = 31.1; SD = 2.43) (P < 0.001 for the test of difference between females and males). At posttest, the adjusted difference between the ARF group and the control group was -0.34 years (P = 0.001) in the female subgroup and -0.12 years (P = 0.548) in the male subgroup. However, post hoc sample size calculation indicates that our study groups were adequately powered for the female subgroup, but not adequately powered for the male subgroup. Statistical interaction tests also suggest that the differences in treatment effects between the two subgroups were not statistically significant (P = 0.298). Hence, we are unable to conclude whether the effects of the intervention differ between women and men (even though the magnitude of the effects was larger in the female subsample).

## Discussion

Findings show that information on age-related fertility decline brought forward university students’ expected timing of family formation without reducing their educational and career expectations. The findings on child-timing ideals are consistent with previous studies that show fertility information reduces desired age for childbearing among university students in the short term [16, 21]. Additionally, the age-related fertility intervention material included a statement that IVF success rates were higher for women who froze their eggs prior to age 35. Findings suggest that female participants who received the age-related fertility decline information intervention increased their expected chances of undergoing social egg freezing by age 25 and by age 30.

Findings also show that information on fertility-related policies had no effects on either family formation or career expectations. These findings contradict our predictions. It could be that fertility-policy information interventions are only effective if they are combined with information on age-related fertility decline. University students’ poor knowledge of age-related fertility decline could have prevented them from understanding how pro-fertility policies could affect their own family formation and career plans. Future studies could examine the effects of providing both types of information simultaneously. Additionally, past research shows that fertility information interventions are more effective if they are tailored and delivered orally [18, 20]. It could be that for fertility policy intervention to be effective, the information provided needs to be tailored to each participant’s particular financial concerns, child-timing desires, and career aspirations. For instance, information on IVF subsidies may be particularly useful for those wishing to delay childbearing for career development, whereas information on baby bonuses and childcare subsidies may be particularly useful for those who wish to have children early but are concerned about the financial costs of child-rearing.

Finally, our findings suggest that both age-related fertility and fertility-related policy information were useful for university students; however, as has been shown previously [19, 20], age-related fertility information induced significantly higher levels of anxiety than both fertility-related policy and the control information (on diabetes).

One strength of our study was the randomized controlled design with a large sample size. We show that a short information brochure on age-related fertility decline with merely six items and 164 words was sufficient to reduce university students’ ideal age of childbearing in the short term. Previous studies, such as Daniluk and Koert [16], used much longer information brochures (10 sections over multiple pages with thousands of words).

Our results suggest that informational interventions to correct inaccurate beliefs about fertility, such as overestimation of IVF success rates [9] or the perception that healthy lifestyles can adequately offset age-related fertility decline, can lead to adjustments of expected timing of childbearing and marriage among university students, without disrupting their educational and career goals, at least in the short term. By addressing informational gaps at earlier ages, individuals and couples are given more time for realistic planning and goal setting. For example, past research suggests that 64% of female university students would not consider oocyte freezing [35], which may reflect a lack of knowledge about the importance of taking first steps at earlier ages.

The effectiveness of such interventions carries implications for public health and reproductive equality. As maternal ages continue to advance, the proportion of births involving assisted reproduction is projected to increase rapidly [36, 37]. Although increased access to assisted reproductive technologies has been shown to offer some promise in raising fertility rates [38] and lowering certain birth risks associated with advanced maternal age [39], assisted reproduction is still an imperfect solution as it cannot fully compensate for the natural decline in fertility with age [40] and may impose psychosocial costs on the patients [41, 42]. As access to and affordability of assisted reproduction improves in the wake of relaxation of local regulations [43], it becomes increasingly important to inform reproductive-aged individuals about the impact of age on fertility and assisted reproduction success rates. Nevertheless, informational interventions should be accompanied by institutional support to provide the desired environments for childbearing at less advanced maternal and paternal ages. Past research suggests that delayed childbearing is often not a deliberate decision but caused by circumstances outside of one’s control, such as relationship status and financial well-being [44]. Women also perceive more negative repercussions of childbearing on their careers [45]. To address these constraints, other forms of interventions should aim at improving work-life balance and reducing economic pressure faced by young families through initiatives such as paid parental leave, subsidized childcare, and family-friendly work environments [46].

### Limitations

We acknowledge several limitations to the present study. One, there was a violation of randomization (i.e., the ARF group comprises a higher share of female students than the other two groups), which happened by chance. However, we presented results using analysis of covariance that adjusted for participant’s gender. The results were not markedly different without the gender adjustment (not shown). Two, participants were recruited from the leading university in Singapore. Our findings may not be generalizable to individuals from a broader socioeconomic background, as past research shows that fertility awareness tends to be more exacerbated among lower SES groups and individuals with less education [12, 14].

Three, the experiment and the interventions were conducted entirely online at a time when on-campus activities and gatherings were limited due to the COVID-19 pandemic. As most students were living off campus and the majority of academic activities were conducted online at the time of the experiment, we expected participants to have answered the questionnaires in isolation. The information was presented in a format that was not conducive to downloads or social media sharing. Nonetheless, we are unable to rule out the possibility that participants in the treated groups may have used screenshots to share general information from the intervention brochure with fellow classmates, either online or in a small group, which would contaminate our experimental design. If such contamination occurred, the estimates we presented here would have underestimated the true effects of the intervention materials on students’ family and career expectations.

Finally, our study was limited in scope in several aspects. Due to budgetary constraints, we did not conduct any pilot study, which could have been used to check for test-retest reliability of the survey questionnaire. Nor could our study design assess the long-term effects of the interventions on childbearing desires and career expectations, which may not be lasting [16]. Future studies could examine the longer-term effects of fertility information on participants’ family formation choices, educational achievements, and career development.

## Conclusion

A short informational brochure with accurate information on age-related fertility decline and IVF success rates could effectively reduce university students’ ideal ages of childbearing. Moreover, at least in the short run, such interventions do not seem to bear any negative consequences on participants’ educational and career expectations.

## Supporting information

S1 File Appendix: Intervention material. (DOC)

S1 Data (ZIP)
