# Supplementary material for: Does information on age-related fertility decline and fertility policies affect university students’ family and career expectations? Evidence from a randomized controlled trial
Source: PLoS One. 2023 Nov 1;18(11):e0287526. doi: 10.1371/journal.pone.0287526 (PMC10619829; doi:10.1371/journal.pone.0287526)
Supplement: S1 File — (DOC) [file pone.0287526.s001.doc]

**Appendix: Intervention Material**

**Intervention material for age-related fertility (ARF) treatment group**

Please spend a few minutes considering the information below.

*Age and fertility*

(1) Age is a more important predictor of women’s fertility than lifestyle choices such as exercising and maintaining a healthy weight.

(2) Older mothers are more likely to experience health problems, such as gestational diabetes and high blood pressure, and have higher chances of miscarriage.

(3) Children born to older mothers are at higher risk of adverse outcomes, including stillbirth, premature birth and low birth weight.

*IVF success rates and side effects*

(1) At the KK Women’s and Children’s Hospital IVF Centre, the probability of live births is around 38% for women aged 30-35 and 19% for women aged 38. Success rates are higher for women who froze their eggs prior to age 35.

(2) The IVF process poses health risks for a woman, including mild to moderate pelvic and abdominal pain, injury to organs near the ovaries, and pelvic infection.

(3) Children conceived by IVF face higher risks of birth defects, especially heart defects and malformations of the urogenital tract.

**Intervention material for fertility policies (FP) treatment group**

Please spend a few minutes considering the information below.

*Assisted reproductive technology treatment subsidies*

(1) At present, there is no age limit for women undergoing procedures for assisted reproduction technology (ART). Prior to 2020, the age limit was set at 45.

(2) The government provides more co-funding for assisted reproductive technologies to married women under age 40. Married women aged below 40 are eligible for co-funds up to 75% of the cost of assisted reproductive technology for six cycles, while married women aged 40 and above are eligible for only two cycles.

(3) In addition, married women aged 40 and above must have attempted assisted reproduction or intra-uterine insemination procedures prior to age 40 in order to be eligible for subsidies.

Other fertility-related policies

(1) Eligibility conditions for first-time homeowners buying a Build-To-Order or resale HDB flat include income ceilings, which are more likely to affect older couples with more established careers. HDB housing grants of up to $80,000 are also subject to income ceilings.

(2) Younger couples with less established careers may be eligible for higher childcare subsidies, which are calculated based on household income.

(3) Under the Baby Bonus Scheme, parents of a Singaporean child receive a total of up to $14,000 in cash gifts and Child Development Account benefits per child for the first and second children, and up to $28,000 for higher parity children.

**Intervention material for control group**

Please spend a few minutes considering the information below.

Diabetes risk factors and treatment

(1) Risk factors for diabetes include obesity, inactivity, hypertension, age, or family history.

(2) Ways to prevent and control diabetes include adopting healthy eating habits, exercising regularly, abstaining from smoking and drinking, and oral medications or insulin injections.

(3) If diabetes is not treated or controlled, it can increase the risk of cardiovascular diseases (e.g. heart attack and stroke, vision conditions, kidney disease, skin infections and other conditions.

Diabetes-related policies

(1) In 2016, the Ministry of Health launched a “War on Diabetes” which seeks to prevent the condition through healthy lifestyles, early detection and intervention, and improved disease management.

(2) There is currently an advertising ban on drinks with very high sugar content, and a mandatory nutrition label for pre-packaged drinks with high sugar or saturated fat content.

(3) Patients with diabetes can withdraw up to $500 from their or their family members’ Medisave accounts annually for treatment, subject to a 15% co-payment in cash.
